# The NLRP3 inflammasome mediates DSS-induced intestinal inflammation in *Nod2* knockout mice

**DOI:** 10.1177/1753425919826367

**Published:** 2019-02-06

**Authors:** Benjamin Umiker, Hyun-Hee Lee, Julia Cope, Nadim J. Ajami, Jean-Philippe Laine, Christine Fregeau, Heidi Ferguson, Stephen E Alves, Nunzio Sciammetta, Melanie Kleinschek, Michael Salmon

**Affiliations:** 1 Merck Research Laboratories, Boston, MA, USA; 2 Diversigen, Inc. Houston, TX, USA; 3 Alkek Center for Metagenomics and Microbiome Research, Baylor College of Medicine, Houston, TX, USA

**Keywords:** NLRP3, NOD2, inflammatory bowel disease, lamina propria, microbiome

## Abstract

Crohn’s disease (CD) is a chronic disorder of the gastrointestinal tract characterized by inflammation and intestinal epithelial injury. Loss of function mutations in the intracellular bacterial sensor NOD2 are major risk factors for the development of CD. In the absence of robust bacterial recognition by NOD2 an inflammatory cascade is initiated through alternative PRRs leading to CD. In the present study, MCC950, a specific small molecule inhibitor of NLR pyrin domain-containing protein 3 (NLRP3), abrogated dextran sodium sulfate (DSS)-induced intestinal inflammation in *Nod2*^−/−^ mice. NLRP3 inflammasome formation was observed at a higher rate in NOD2-deficient small intestinal lamina propria cells after insult by DSS. NLRP3 complex formation led to an increase in IL-1β secretion in both the small intestine and colon of *Nod2*ko mice. This increase in IL-1β secretion in the intestine was attenuated by MCC950 leading to decreased disease severity in *Nod2*ko mice. Our work suggests that NLRP3 inflammasome activation may be a key driver of intestinal inflammation in the absence of functional NOD2. NLRP3 pathway inhibition can prevent intestinal inflammation in the absence of robust NOD2 signaling.

## Introduction

Inflammatory bowel disease (IBD) is a heterogeneous group of diseases characterized by aberrant inflammation of the gastrointestinal tract. IBD is initiated by various genetic and environmental factors and patients present a heterogeneous set of symptoms and wide-ranging response to current treatments.^1^^,^^2^ Many studies investigating the role of microbial communities and microbial diversity in the intestinal tract of IBD patients have been conducted.^3^ It is clear that the intestinal microbiome plays an important role in the establishment of a homeostatic relationship with the host immune system.^4^

Polymorphisms of multiple genes involved in the recognition of microbial products are associated with an increased risk for the development of IBD. Mutations in NOD2 is highly associated with Crohn’s disease (CD).^5^^,^^6^ NOD2 is an intracellular innate PRR which is critical for the response to particular strains of bacteria. Recognition of muramyl dipeptide (MDP) by NOD2 initiates a signaling cascade that is dependent on both receptor-interacting serine/threonine-protein kinase 2 (RIPK2) and NF-kB to produce pro-inflammatory cytokines and antimicrobial peptides.^7^

Many studies using mouse models of IBD have been performed to further understand the mechanisms by which NOD2 affects the pathogenesis of CD. There are conflicting reports about the susceptibility of *Nod2*^−/−^ mice to dextran sodium sulfate (DSS)-induced colitis. Multiple studies have shown an increased sensitivity to DSS induced colitis in *Nod2*^−/−^ mice,^8^^,^^9^ while others have observed no difference between NOD2 deficient animals and wild type mice exposed to DSS.^10^

NLRP3 is a member of the NLR family that is responsible for inflammatory responses. The NLRP3 inflammasome is a multi-protein complex consisting of NLRP3, the adaptor molecule apoptosis-associated speck-like protein (ASC), and procaspase-1. Upon recognition of a diverse set of signals the NLRP3 inflammasome oligomerizes into a scaffold that activates pro-caspase-1. In turn the activated caspase-1 cleaves pro-IL-1 and pro-IL-18, which leads to the secretion of the active forms of these cytokines. There is conflicting evidence for the role of NLRP3 in the pathogenesis of intestinal inflammation. *Nlrp3*^−/−^ mice have been shown to be both more susceptible to and protected against DSS- and TNBS-induced intestinal inflammation in different studies.^11^^,^^12^ MMC950, a specific inhibitor of NLRP3 inflammasome formation,^13^ was shown to attenuate inflammation in a spontaneous model of chronic colitis, in which a mutation in the Muc2 gene results in aberrant Muc2 secretion and inflammation.^14^

In the absence of robust NOD2 signaling it is probable that other PRRs drive the inflammation that affects CD patients. Here we investigate whether NLRP3-driven inflammation is critical for DSS-induced intestinal damage using MCC950. In the present study, MCC950 was found to attenuate DSS-induced disease severity in *Nod2^−^*^/^*^−^* mice, but not wild type mice. This is due to an increase in NLRP3 inflammasome formation and IL-1β production in the intestinal tract of *Nod2^−^*^/^*^−^* mice compared with wild type mice.

## Material and methods

### Mice and DSS model

Littermate wild type and *Nod2*^−/−^ mice at 8 wk of age were used in all studies and the mice were breed at Taconic. Wild type and *Nod2*^−/−^ littermates were separated and shipped in separate cages after genotyping. They were kept in the local mouse facilities for 2 wk before DSS experiments were run. Age-matched female mice were exposed to 2% DSS in the drinking water for 9 d. MCC950 was incorporated into the chow at 1 mg/kg of food and administered at d 0 of the study. The weight of the food was monitored throughout the study. No differences were found in consumption of the medicated chow and normal chow.

Body weight, the presence of occult or gross blood per rectum, and stool consistency were determined by two investigators blinded to the treatment groups. A scoring system was applied to assess diarrhea and the presence of occult or overt blood in the stool. Changes of body weight are indicated as loss of baseline body weight as a percentage. *Post mortem*, the colon and small intestine was removed washed with HBBS and either flash frozen for later homogenization or put on ice for isolation of lamina propria cells.

The following scoring system was used to determine disease activity index (DAI), as described in Kim et al.^15^ Body weight loss percent relative to d 1: 0 = no weight loss, 1 = 1–10%, 2 = 10–15%, 3 = 15–20%, 4 = >20%. Stool consistency was scored daily as follows: 0 = normal, 1 = soft but still formed, 2 = very soft, 3 = diarrhea/rectal prolapse <1 cm, 4 = diarrhea/rectal prolapse >1 cm. Bleeding was assessed daily by two methods: a positive guaiac occult blood test or visually observing blood in the stool. Feces were collected and smeared directly on to paper hemoccult slides for testing presence of blood. Bleeding was scored as follows: 0 = normal stool, 1 = light positive hemoccult, 2 = dark positive hemoccult, 3 = blood visibly present, 4 = gross rectal bleeding. DAI was determined by adding the three scores.

MCC9950 was used to inhibit NLRP3 inflammasome formation *in vivo* by incorporating the compound in the chow of mice. The synthesis of MCC9950 and incorporation into the chow occurred in house. The chow was synthesized with 1 g of MCC9950 per 1 kg of chow. This translates into an approximate dose of 150 mg of compound per kg of body weight per d. The amount of food consumed every 2 d was measured and no significant differences were observed between groups (data not shown). The approximate dose was ascertained from previous pharmacokinetic study using MCC950 in wild type mice, in which no toxicity was observed (data not shown).

For microbiome analysis, fecal pellets were collected from each mouse using the clean catch method at d 0, 2, and 7 after treatment. In addition, 30 ileal small intestine tissue and 30 colon samples were collected from DSS exposed and untreated and wild type and *Nod2*^−/−^ mice at the end of the study.

### 16S rDNA sequencing

Bacterial DNA from feces was extracted using MO BIO PowerMag Microbiome DNA Isolation Kit (MO BIO Laboratories). Similarly, bacterial DNA from tissues was extracted using MoBio Tissue and Cell DNA extraction kit. Extracted DNA was subjected to 16S rDNA V4 region was PCR amplification sequenced in the MiSeq platform (Illumina) using 2 × 250 bp chemistry. The primers used for amplification contain adapters for MiSeq sequencing and single-end barcodes allowing pooling and direct sequencing of PCR products.^16^ Sequence read pairs were demultiplexed based on the unique molecular barcodes, and reads were merged using USEARCH v7.0.1090,^17^ allowing zero mismatches and a minimum overlap of 50 bases. Merged reads were trimmed at first base with Q5. In addition, a quality filter was applied to the resulting merged reads and reads containing above 0.05 expected errors were discarded. Resulting reads were clustered into operational taxonomic units (OTUs) at a similarity cutoff value of 97% using the UPARSE algorithm.^18^ OTUs were mapped to an optimized version of the SILVA Database,^19^ containing only the 16S v4 region to determine taxonomies. Abundances were recovered by mapping the demultiplexed reads to the UPARSE OTUs. A custom script constructed a rarefied OTU table from the output files generated in the previous two steps for downstream analyses of alpha-diversity, beta-diversity,^20^ and phylogenetic trends.

### Cytokine and mRNA profiling

After weighing the tissue, colon and small intestine homogenates were obtained using the tissue extraction reagent with protease inhibitor (Invitrogen) and then using a tissue homogenizer. Tissue fragments were removed by centrifugation (10,864 *g*, 5 min).

Total bone marrow was isolated from two femurs per mouse. EasySep mouse Monocyte Enrichment kit was used to isolate a high percentage of monocytes from the bone marrow. Enriched monocytes at 50,000 cells per well were plated in 96 well plates and treated with mGM-CSF at 50 ng/ml for 6 d. Non- and semi-adherent cells were washed away. Bone marrow derived macrophages (BMDM) were then treated with media containing multiple treatments. Twenty-four h after treatment supernatants were collected for cytokine analysis and 20 µl of RNeasy Lysis Buffer (Qiagen) buffer + 1% β-mercaptoethanol was added to lyse cells for mRNA profiling by NanoString nCounter® Systems using the mouse Immunology kit through the protocol provided by the manufacturer. Cytokines from supernatants and homogenates were determined using Mesoscale discovery 10plex Mouse pro-inflammatory kit through the protocol provided by the manufacturer.

### FACS analysis

Small intestinal lamina propria (SILP) cells were isolated using a Lamina Propria Dissociation Kit (Miltenyi Biotec) and analyzed using a BD FACS CANTO II. The following primary Abs were used: anti-mouse CD45 A647 (Biolegend 30-F11), anti-mouse CD11b PE-Cy7 (Biolegend), anti-mouse Ly-6G PerCP-Cy5.5 (Biolegend), anti-mouse F4/80 PE (Biolegend).

### Duolink PLA assay

THP-1 cells, macrophages derived from the bone marrow, or SILP cells were plated overnight in a 96 well glass bottom plate in DMEM with 10% FBS. This was followed by 24 h by a pretreatment of LPS at 5 µg/ml, and then followed by 24 h of nigericin at 5 µg/ml with an without MCC950 at 1 µM. Cells were fixed and permeabilized using the Image-It Fix-Perm kit (life Technologies). Primary Abs were added at 1:500: rabbit anti-ASC/TMS1 (Sigma S1B8731) and goat anti-NLRP3/CIAS1 (Sigma SAB2501362) and incubated for 2 h at 37°C. The PLA probes were added at 1:5: PLA probe anti-goat minus (DUO92006) and PLA probe anti-rabbit plus (Duo92002) for 1 h at 37°C. Ligation buffer ligase was added at 1:40 and added to cells for 30 min at 37°C. Amplification buffer polymerase was added at 1:80 and added to cells for 100 min at 37°C. Cells were washed and mounted with DAPI (4′,6-diamidino-2-phenylindole). Immune fluorescence staining was analyzed on an Opera high content screening microscope.

### Statistical analysis

Data are expressed as means ± SEM. Statistical significance of differences between treatment and control groups was determined by ANOVA. Differences were considered statistically significant at *P* < 0.05.

Data analysis and visualization of microbiome communities was conducted in R, utilizing the phyloseq package to import sample data and calculate alpha- and beta-diversity metrics.^21^ Significance of categorical variables were determined using the non-parametric Mann–Whitney test for two category comparisons or the Kruskal–Wallis test when comparing three or more categories. Principal coordinate plots employed the Monte Carlo permutation test to estimate *P* values. All *P* values were adjusted for multiple comparisons using the FDR algorithm.

## Results

### Nod2^−/−^ mice are more susceptible to DSS-induced colitis than wild type mice

**Figure 1.**
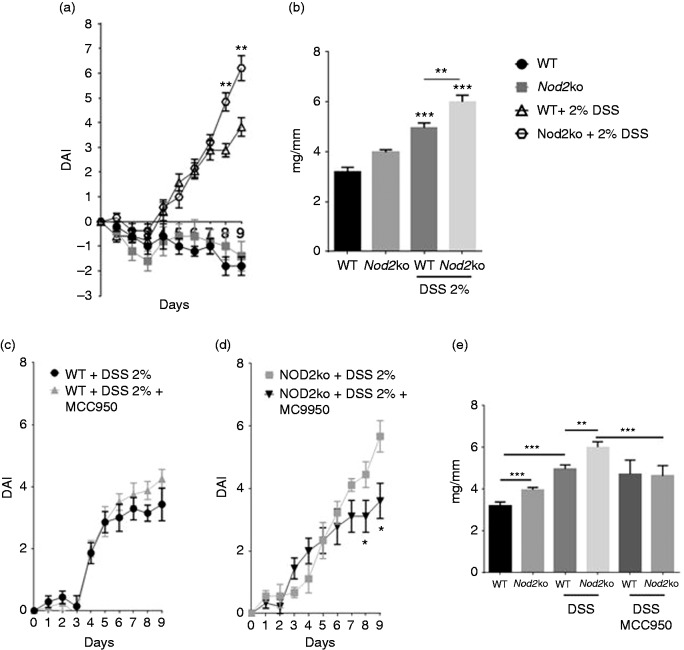
Eight wk-old C57BL/6 and NOD2ko littermate controls were given 2% DSS in the drinking water for 9 d, there were at least 10 mice per group. (a) Disease activity index (DAI) scored by the addition of stool consistency score, occult fecal blood score and body weight score, (b) Colon weights (mg) divided by the length of colon (mm) on d 9 of DSS treatment, (c) DAI of wild type mice treated with MCC950, (d) DAI in *Nod2*^−/−^ mice treated with MCC950 and (e) Colon weights (mg) divided by the length of colon (mm) on d 9 of DSS treatment. Statistical analysis is based on a one-way ANOVA. *P < 0.05, **P < 0.01, ***P < 0.001, and ****P < 0.0001.

Littermate *Nod2*^−/−^ and wild type mice were exposed to 2% DSS to monitor disease under our specific housing conditions. *Nod2*^−/−^ mice lost significantly more weight over the course of DSS treatment compared with wild type controls (see Supplemental Figure 1a online). *Nod2*^−/−^ mice lost on average 4.1% (*P* = 0.03) more body weight after DSS treatment on d 9 compared with wild type litter mate controls exposed to DSS. Similarly, *Nod2*^−/−^ mice had significantly looser stools on d 9 with an average fecal consistency score of 2.4 compared with an average score of 1.9 for wild type mice exposed to DSS (*P* = 0.01). After 8 d of 2% DSS exposure, bleeding was observed in 70% of *Nod2*^−/−^ mice and gross rectal bleeding in 60% of *Nod2*^−/−^ mice compared with wild type controls in which only 10% of mice had visible fecal blood. *Nod2*^−/−^ mice had a fecal blood score of 3.0 on d 9 of DSS exposure compared with 1.5 for wild type controls (*P* < 0.0001) (Supplemental Figure 1c). The DAI was recorded using method described in the ‘Materials and Methods’ in Edgar et al (2013).^22^ *Nod2*^−/−^ mice had significantly higher DAI scores than wild type mice after 8 and 9 d of 2% DSS exposure. The DAI for *Nod2^−^*^/^*^−^* mice was 93% higher (*P* = 0.003) on d 8 and 63% higher (*P* = 0.02) on d 9 compared with wild type mice exposed to DSS. The ratio of colon weights to colon length was measured as a marker of colonic inflammation. *Nod2*^−/−^ mice had a 21% higher colon weight:length ratio (*P* = 0.003) after treatment with DSS for 9 d compared with control mice (*P* = 0.03) (Figure 1b).

### The specific NLRP3 inhibitor, MC9950, attenuates disease severity in Nod2^−/−^ mice, but not wild type mice

To test whether the NLRP3 inflammasome plays a role in DSS induced intestinal inflammation, the NLRP3 specific small molecule inhibitor MC9950 was administered *in vivo*. MC9950 had no significant effect on the DAI in wild type mice exposed to 2% DSS for 9 d (Figure 1c). *Nlrp3^−^*^/^*^−^* mice had similar DAI scores to wild type mice after 3% DSS exposure for 6 d (Supplemental Figure 7). Neither NLRP3 deficiency nor MCC950 treatment in wild type mice had an effect on colon weight:length ratio (Figure 1e and supplemental Figure 7) However, in *Nod2*^−/−^ mice MC9950 significantly attenuated the DSS-induced disease severity. The average DAI on d 9 was 3.9 in *Nod2^−^*^/^*^−^* mice treated with MCC950 compared with 5.7 in *Nod2^−^*^/^*^−^* mice unexposed to MCC950 (Figure 1d). The weight to length ratio was 6.0 mg/mm in *Nod2^−^*^/^*^−^* mice treated with MCC950 compared with 4.6 mg/mm in *Nod2^−^*^/^*^−^* mice without the compound (Figure 1e).

THP-1 cells differentiated using PMA treated with nigericin, an NLRP3 agonist, produced high levels of IL-1β. PMA differentiated THP-1 cells were pre-treated for 30 min with MC9950 followed by the addition of nigericin for 24 h. A concentration-dependent inhibition of IL-1β secretion by MC9950 was observed in PMA differentiated THP-1 cells (Supplementary Figure 2A). Other cytokines and chemokines induced by nigericin in THP-1 cells were not blocked by MC9950, including IL-8 (Supplemental Figure 5). A Duolink proximity ligation assay developed to detect the formation of the NLRP3 inflammasome by immune fluorescence was performed on THP-1 cells. The assay allows for the detection of NLRP3 and ASC complex formation by detecting proximity of the two proteins through immunofluorescence. The NLRP3/ASC complex was detected in PMA differentiated cells after 24 h of nigericin treatment at 5 µg/ml (Supplementary Figure 2B) and its formation was blocked by MC9950 at 1 µg/ml (Supplementary Figure 2B).

**Figure 2.**
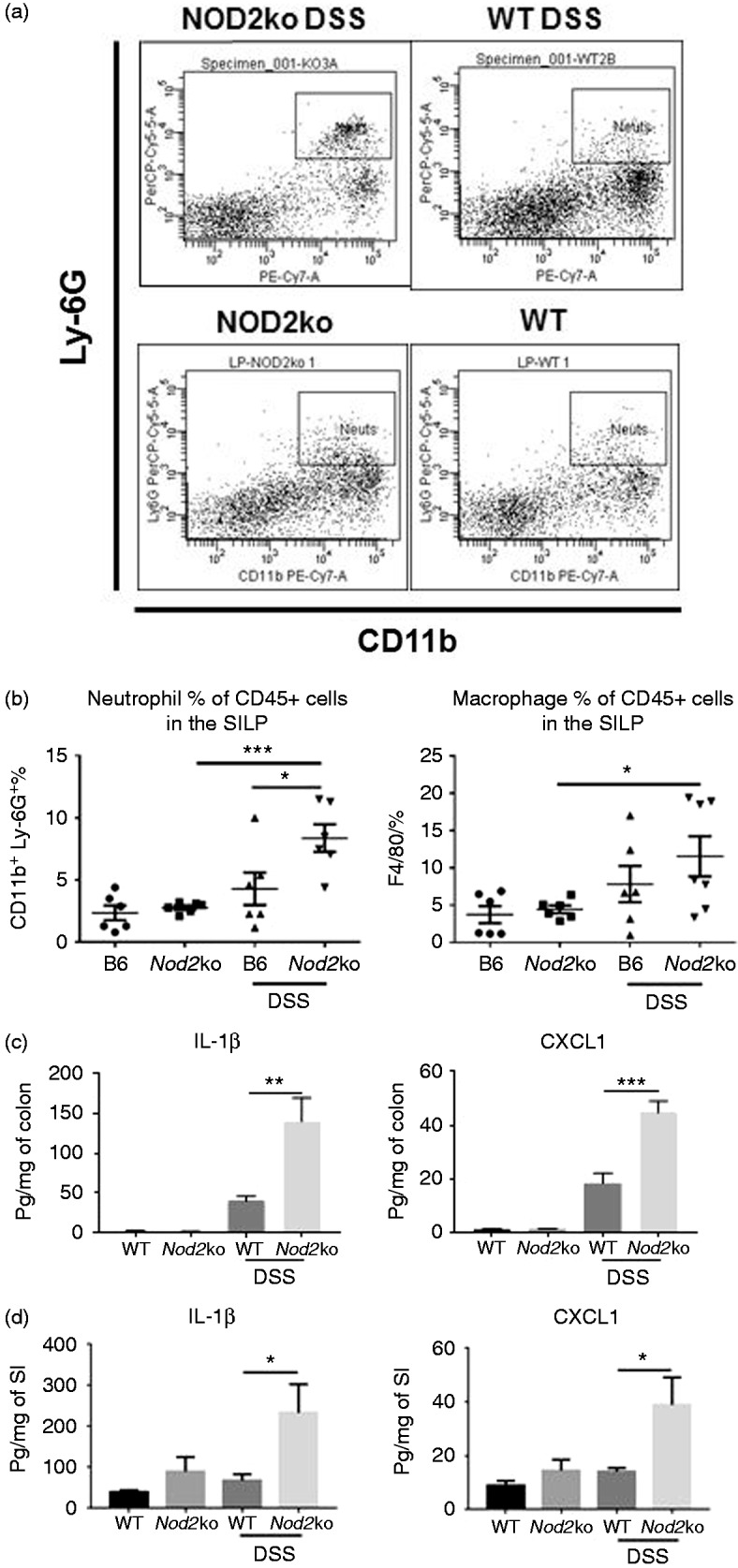
CD45+ cells were isolated from the lamina propria of the small intestine and stained for CD11b+/Ly-6G+ neutrophils and F4/80+ macrophages. (a) Example plots of neutrophil staining from DSS-treated and untreated wild type and Nod2ko animals on d 7 of DSS treatment, (b) Analysis of the neutrophil and macrophage populations in the SILP with 6 mice per group on d 9 of DSS treatment. Concentrations of IL-1β and CXCL1 in colon homogenates (c) or small intestine (d) divided by the total protein concentration of the homogenates from at least 6 mice per group. Statistical analysis is based on a one-way ANOVA. *P < 0.05, **P < 0.01, ***P < 0.001, and ****P < 0.0001.

### Nod2^−/−^ mice have increased expression of inflammatory cytokines and increased number of inflammatory cells in the intestine compared to wild type controls

SILP from *Nod2*^−/−^ and wild type mice were isolated to measure the abundance of inflammatory cells in the mucosal layer of the small intestine. There was a higher percentage of CD45+/CD11b+/Ly-6G+ neutrophils in the small intestine of *Nod2*^−/−^ mice after 9 d of 2% DSS exposure compared with wild type mice exposed to DSS (*P* = 0.04) and compared with *Nod2*^−/−^ mice not exposed to DSS (*P* = 0.0005) (Figure 2a and b) There was also a significantly higher percentage of F4/80+ macrophages in the SILP of *Nod2*^−/−^ mice exposed to DSS and untreated *Nod2*^−/−^ mice with 11.5% in the SILP of DSS-treated *Nod2*^−/−^ mice compared to 4.5% in untreated *Nod2*^−/−^ mice (*P* = 0.036) (Figure 2b).

Colon and small intestinal homogenates were obtained after 7 d of DSS exposure from wild type and *Nod2*^−/−^ mice and total protein levels and cytokine and chemokine concentrations were determined. There was an increase in pro-inflammatory cytokines and chemokines in the colon of both wild type and *Nod2^−^*^/^*^−^* mice treated with DSS, including IL-1β, CXCL1, TNF-α, and IL-6 in the colon (Figure 2c and Supplemental Figure 1). After DSS exposure, *Nod2*^−/−^ mice had a higher concentration of IL-1β and CXCL1 in the colon compared with wild type mice (Figure 2c). Unlike in the colon, there was no significant increase in the cytokines measured from the small intestine in wild type mice exposed to DSS (Figure 2d). However, *Nod2^−^*^/^*^−^* mice had a significant two- to threefold increase in IL-1β and CXCL1 in the small intestine after DSS treatment (Figure 2d).

### Changes in the composition and structure of microbial communities of the small intestine in Nod2^−/−^ mice after treatment with DSS

**Figure 3.**
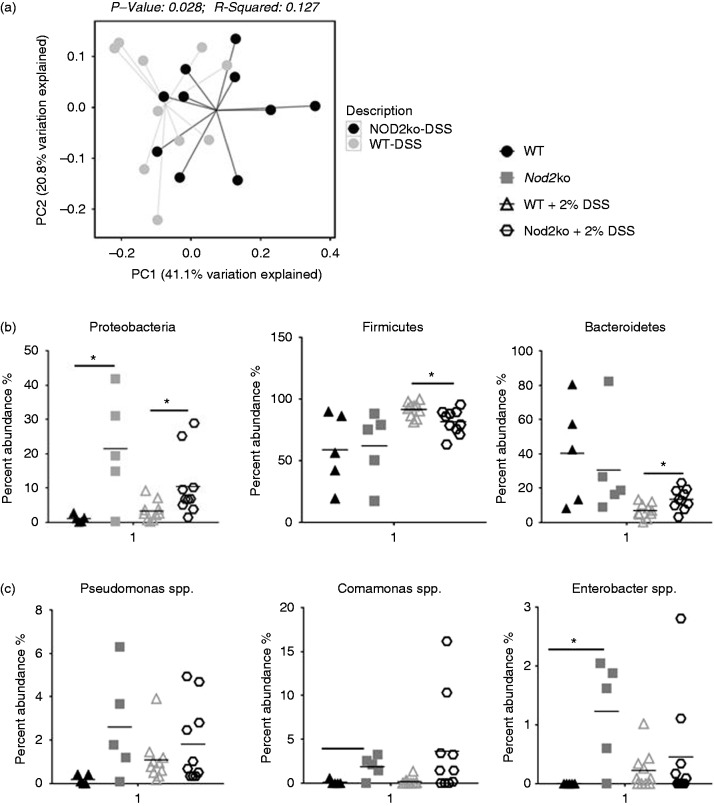
(a) PCoA plot of weighted UniFrac distances of small intestinal samples after DSS treatment comparing wild type and NOD2^−/−^ mice after 9 d, (b) Relative abundance of bacterial phyla in the small intestine of mice treated with DSS after 9 d and (c) Relative abundance of bacterial selected bacterial genera identified in the small intestine of mice treated with DSS.

The microbial composition and structure between the small intestine, the colon and the feces were measured in both wild type and *Nod2^−^*^/^*^−^* mice before and after DSS-induced intestinal inflammation. DSS induced significant compositional changes in the microbiota of the small intestine, colon and feces in both wild type and *Nod2^−^*^/^*^−^* mice (Supplemental Figure 8). Samples from the small intestine from *Nod2*^−/−^ and WT mice exposed to DSS showed a significantly different community structure as evidenced by distinct clusters in a PCoA plot using a weighted UniFrac analysis (Figure 3a). No significant differences in microbial community structures were observed in the feces of Nod2ko mice compared with wild type (Supplemental Figure 10) There were no differences in bacterial richness between genotypes or treatment groups (data not shown). The relative abundance of the taxa identified determined by mapping the OTUs to the SILVA database was evaluated and significant differences were observed in the abundance of major phyla after DSS treatment including an increase in Proteobacteria and Bacteroidetes and a decrease in Firmicutes in the terminal ileum compared to wild type mice after exposure to DSS for 9 d (Figure 3b). Analysis at the genus level revealed significantly higher abundance in *Nod2^−^*^/^*^−^* small intestine of *Pseudomonas*, *Comamonas*, and *Enterobacteriaceae* (Figure 3c).

### Inflammasome formation and IL-1β secretion is increased in Nod2^−/−^ mice and is blocked by MC9950

**Figure 4.**
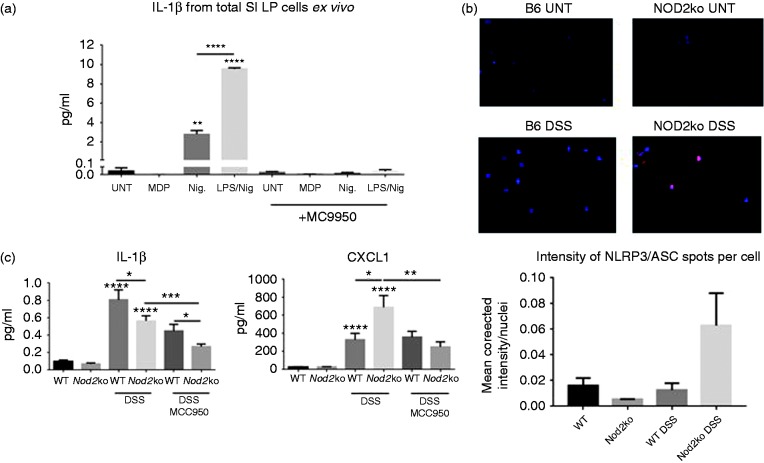
(a) *Ex vivo* analysis of SILP cells. Cells isolated from DSS-treated animals with and without the treatment with MCC950. *Ex vivo* cells are treated for 24 h with nigericin or LPS + nigericin and supernatants are measured for IL-1β secretion, (b) Inflammasome formation as measured by ASC/NLRP3 Duolink PLA staining in SILP. Intensity of ASC/NLRP3 signal per nuclei. Average value of 3 mice per group, at least 500 cells analyzed per mouse and (c) IL-1β and CXCL1 concentrations in the serum of mice, at least 8 mice per group. Statistical analysis is based on a one-way ANOVA. *P < 0.05, **P < 0.01, ***P < 0.001, and ****P < 0.0001.

Lamina propria cells were isolated from the small intestine of wild type mice exposed to 2% DSS for 9 d. Lamina propria cells were stimulated *ex vivo* for 24 h with nigericin either with or without LPS. Lamina propria cells from the small intestine produced IL-1β after nigericin stimulation. However, *ex vivo* stimulation by nigericin of cells isolated from mice treated with MC9550 did not secrete IL-1β (Figure 4a). This suggested a high level of target engagement in the lamina propria by MC9950 to NLRP3. ASC/NLRP3 complexes were detected in SILP cells from *Nod2*^−/−^ mice exposed to 2% DSS at a higher rate than in WT mice by Duolink proximity ligation assay (Figure 4b). Using a high content screening confocal microscope, the intensity of NLRP3/ASC inflammasome formation was measured per cell isolated from the SILP from three mice per group. At least 1000 cells were analyzed per mouse. Inflammasome formation was observed in *Nod2^−^*^/^*^−^* mice exposed to DSS at a higher rate than in wild type mice exposed to DSS (Figure 4b). MCC950 affected the cytokine and chemokine profile in the sera of *Nod2^−^*^/^*^−^* mice (Figure 4c and Supplemental Figure 6). Circulating CXCL1 was significantly higher in DSS-treated mice compared to untreated mice. Serum levels of CXCL1 were significantly higher in *Nod2^−^*^/^*^−^* mice exposed to 2% DSS compared to wild type mice (Figure 4c). These high levels of circulating CXCL1 in *Nod2^−^*^/^*^−^* mice were not observed in MCC950 treated *Nod2^−^*^/^*^−^* mice exposed to 2% DSS (Figure 4c). DSS exposure increased both TNF-α and IFN-γ in the serum in wild type and *Nod2^−^*^/^*^−^* mice (Supplemental Figure 6) while treatment with MCC950 reduced the amount of circulating IL-1β in DSS-treated mice of either phenotype (Figure 4c).

**Figure 5.**
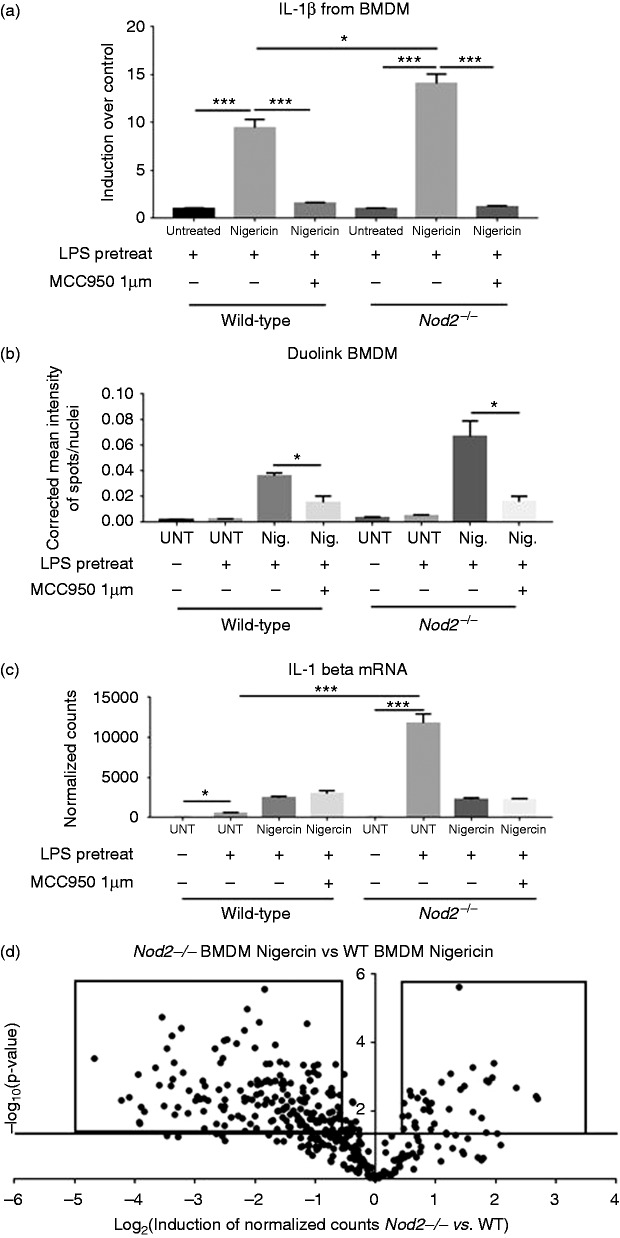
(a) IL-1β from BMDMs stimulated *ex vivo* by nigericin at 5 μg/ml after pre-treatment with LPS at 5 μg/ml, MCC950 added at 1 μM. IL-1β is measured in pg/ml, here is it displayed as fold induction over the LPS pretreated followed by untreated media, (b) Inflammasome formation as measured by ASC/NLRP3 Duolink staining in the BMDM. Corrected mean intensity of ASC/NLRP3 signal per nuclei. Forty-nine fields analyzed per well, three wells analyzed per group, and shown as average of three wells, (c) mRNA IL-1β transcript levels normalized to housekeeping genes from BMDMs and (d) Volcano plot of 800 genes from the Nanostring mouse immunology panel. Compares BMDM treated with nigericin and pre-treated with LPS from Nod2^−/−^ mice versus wild type mice. Horizontal line represents a *P* value of 0.05, dots above the line are considered significant inductions. Dots in boxes are up-regulated and down-regulated genes listed in Supplemental Figure 9. Statistical analysis is based on a one-way ANOVA. *P < 0.05, **P < 0.01, ***P < 0.001, and ****P < 0.0001.

BMDM were isolated from *Nod2^−^*^/^*^−^* mice and wild type mice. *Ex vivo* treatment with nigericin after pre-stimulation with LPS induced IL-1β secretion from BMDM. In wild type cells IL-1β was induced nine-fold over baseline by nigericin while in *Nod2^−^*^/^*^−^* cells IL-1β was induced 14-fold (Figure 5a). In cells from both genotypes 1 μM MC9950 reduced the secretion of IL-1β by nigericin to baseline levels (Figure 5a). Using Duolink PLA it was found that in *Nod2^−^*^/^*^−^* BMDM there had a higher level of NLRP3 inflammasome formation than in wild type BMDM (Figure 5b). IL-1β mRNA transcription was induced by LPS in wild type BMDM; however, the level of mRNA transcripts of IL-1β upon LPS stimulation of *Nod2^−^*^/^*^−^* BMDM was 23.5 times higher compared with LPS-treated wild type BMDM (Figure 5c). Nigericin modulated the expression of IL-1β transcripts, but MCC950 had no effect on the mRNA transcript levels (Figure 5c). There were significant differences in transcript levels of many genes comparing *Nod2^−^*^/^*^−^* and wild type BMDM after stimulation with LPS and nigericin (Figure 5d and Supplemental Figure 11). This indicates that the presence of NOD2 affects the downstream processes after stimulation of the TLR and NLRP3 pathways.

## Discussion

An increased susceptibility to intestinal inflammation in *Nod2^−^*^/^*^−^* animals was abrogated by the treatment of a specific NLRP3 inhibitor, MCC950. There was a corresponding increase in formation of the NLRP3 inflammasome in SILP cells after treatment with DSS in *Nod2^−^*^/^*^−^* mice. *Nod2^−^*^/^*^−^* BMDM produce high amounts of IL-1β when stimulated by an NLRP3 agonist, nigericin. Signaling through NOD2 in macrophages has been linked previously to an increase in NLRP3 inflammasome driven IL-1β secretion.^23^^,^^24^ NOD2 signaling leads to a WNT dependent signaling cascade that leads to NLRP3 inflammasome activation through XIAP.^25^ Our work demonstrates that in the absence of NOD2 the NLRP3 inflammasome is formed and drives increased intestinal inflammation upon injury by DSS treatment and that this inflammation is reversible with a small molecule specific inhibitor of NLRP3.

Early work on CD-associated NOD2 genetic variants focused on whether these mutations increased or decreased the signaling capacity of NOD2.^26^ A seminal study demonstrated that IL-1β drives colitis in mice with the mouse NOD2 variant equivalent to the human CD variant *3020insC*.^27^ The authors concluded that this NOD2 variant increased the capacity for NOD2 to induce IL-1β directly. However, NOD2 variants associated with increased CD risk are loss of function alleles. In our study we observed that *Nod2^−^*^/^*^−^* mice were more susceptible to DSS-induced inflammation and that this increased susceptibility was dependent on NLRP3-induced IL-1β production. We would posit that earlier studies with the NOD2 CD variant in murine models had increased NLRP3 activation leading to the observed IL-1β induced inflammation. Interestingly, knockdown of NLRP3 in an intestinal epithelial cell line showed subsequent increased activation through NOD2,^28^ demonstrating cross-talk between these two pathways. We observed NLRP3-dependent IL-1β in the colon and small intestine, *in vivo*, was increased in the absence of NOD2. Activation of the NLRP3 pathway is responsible for the increased severity of disease in DSS-treated *Nod2^−^*^/^*^−^* mice.

Further work is needed to fully understand the mechanistic connection between a loss of NOD2 signaling and increases in NLRP3 formation and inflammasome activation. There is evidence that NLRP3 expression is critical in IL-1β secretion after stimulation with NOD2 agonist, MDP.^29^^,^^30^ MDP stimulation leads to a WNT dependent signaling cascade that leads to NLRP3 inflammasome activation through XIAP that can exacerbate arthritis in a mouse model.^31^ One study found *in vitro*, that IL-1β secretion by MDP is dependent on both NOD2 and NLRP3 expression.^32^^,^^33^ However, paradoxically it has been found that MDP can stimulate IL-1β secretion in the absence of NOD2 signaling *in vivo*.^27^ Along with our data this argues that in the absence of NOD2, MDP-driven NLRP3 inflammasome activation could be increased. Potentially, the availability of MDP for NLRP3 activation is increased in the absence of NOD2. Future work with CD patient cells could reveal if loss of function alleles of NOD2 have increased NLRP3 inflammasome activation in human disease.

Significant differences in the intestinal microbiome of *Nod2*-deficient mice compared with wild type mice,^9^^,^^20^^,^^34–36^ and human CD patients with mutations of NOD2 have been reported.^37^ It is still an open question whether dysbiosis of the intestinal microbiome can trigger CD or if it is potentially one of many factors which drive disease progression. One study in mice demonstrated that NOD2 is responsible for the prevention of the expansion of species of bacteria in the intestine, *Bacteroides vulgatus*. *B. vulgatus* was shown to be responsible for *Nod2*^−/−^ mice increased sensitivity to small intestinal damage and inflammation.^38^ Another report demonstrated that in *Nod2*^−/−^ mice there was a transmissible dysbiotic intestinal microbiota which conferred increased sensitivity to DSS-induced colitis and DSS/AOM induced colorectal cancer.^9^ However, other groups have found no difference in the microbial communities in the feces or in mucosal scrapings of *Nod2*-/- mice.^39^ The varied results implicating the microbiome and increased inflammation in *Nod2*^−/−^ mice are often attributed to differences in housing and breeding conditions, both temporal and locational differences in sampling, or differences in sequencing methodology. In our report we observed significant differences in the microbiome of the small intestine between *Nod2*^−/−^ and wild type mice before and after treatment with DSS, who were F2 littermate controls. No differences where observed in the colon or feces. We observed an increase in *Proteobacteria* in the small intestine of *Nod2*^−/−^ mice that correlates with an increase in disease severity. Similarly, *Proteobacteria* were found at a higher rate in the small intestine of patients with CD.^40^

There are conflicting reports about the role of NLRP3 in intestinal inflammation. Others have demonstrated that NLRP3 plays an important role in the induction of colitis in DSS-treated mice and a spontaneous model of colonic inflammation,^11^^,^^14^ while others have observed a protective effect for NLRP3.^41^ In the present study, MCC950 was shown to have efficacy in attenuating DSS intestinal inflammation, but only in the highly susceptible *Nod2*^−/−^ mice. This supports the role of NLRP3 as an important driver of inflammation in damaged intestines. There was an increase of NLRP3 dependent IL-1β production in both the small intestine and colon leading to increased inflammation and disease severity in *Nod2*^−/−^ mice upon DSS exposure. NLRP3-induced inflammation may be an important pathway in CD patients with polymorphisms of the NOD2 gene and our studies would suggest that NLRP3 targeting therapies have potential efficacy in this subset of patients.

## Supplemental Material

Supplemental Figures - Supplemental material for The NLRP3 inflammasome mediates DSS-induced intestinal inflammation in *Nod2* knockout mice Click here for additional data file. Supplemental material, Supplemental Figures for The NLRP3 inflammasome mediates DSS-induced intestinal inflammation in *Nod2* knockout mice by Benjamin Umiker, Hyun-Hee Lee, Julia Cope, Nadim J. Ajami, Jean-Philippe Laine, Christine Fregeau, Heidi Ferguson, Stephen E Alves, Nunzio Sciammetta, Melanie Kleinschek and Michael Salmon in Innate Immunity

## Supplemental Material

Supplemental Table - Supplemental material for The NLRP3 inflammasome mediates DSS-induced intestinal inflammation in *Nod2* knockout mice Click here for additional data file. Supplemental material, Supplemental Table for The NLRP3 inflammasome mediates DSS-induced intestinal inflammation in *Nod2* knockout mice by Benjamin Umiker, Hyun-Hee Lee, Julia Cope, Nadim J. Ajami, Jean-Philippe Laine, Christine Fregeau, Heidi Ferguson, Stephen E Alves, Nunzio Sciammetta, Melanie Kleinschek and Michael Salmon in Innate Immunity
