# Supplementary material for: The NLRP3 inflammasome mediates DSS-induced intestinal inflammation in Nod2 knockout mice
Source: Innate Immun. 2019 Feb 6;25(2):132–43. doi: 10.1177/1753425919826367 (PMC6830860; doi:10.1177/1753425919826367)
Supplement: Supplemental Figures - Supplemental material for The NLRP3 inflammasome mediates DSS-induced intestinal inflammation in Nod2 knockout mice [file Supplemental_Figures.pdf]

Supplemental 1

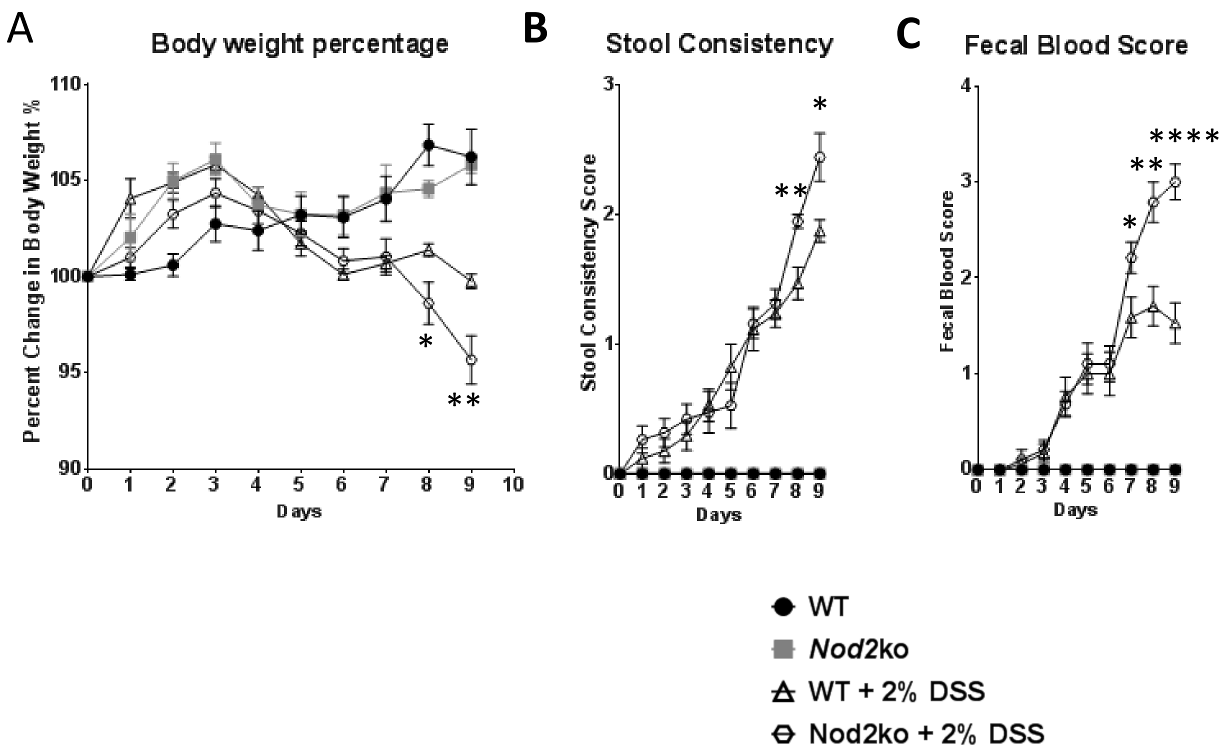

A. Daily percentage of body mass loss or gain compared to body mass on d 0 of the study. **B., C.** Stool consistency and occult fecal blood in the stool scored daily.

Supplemental Figure 2

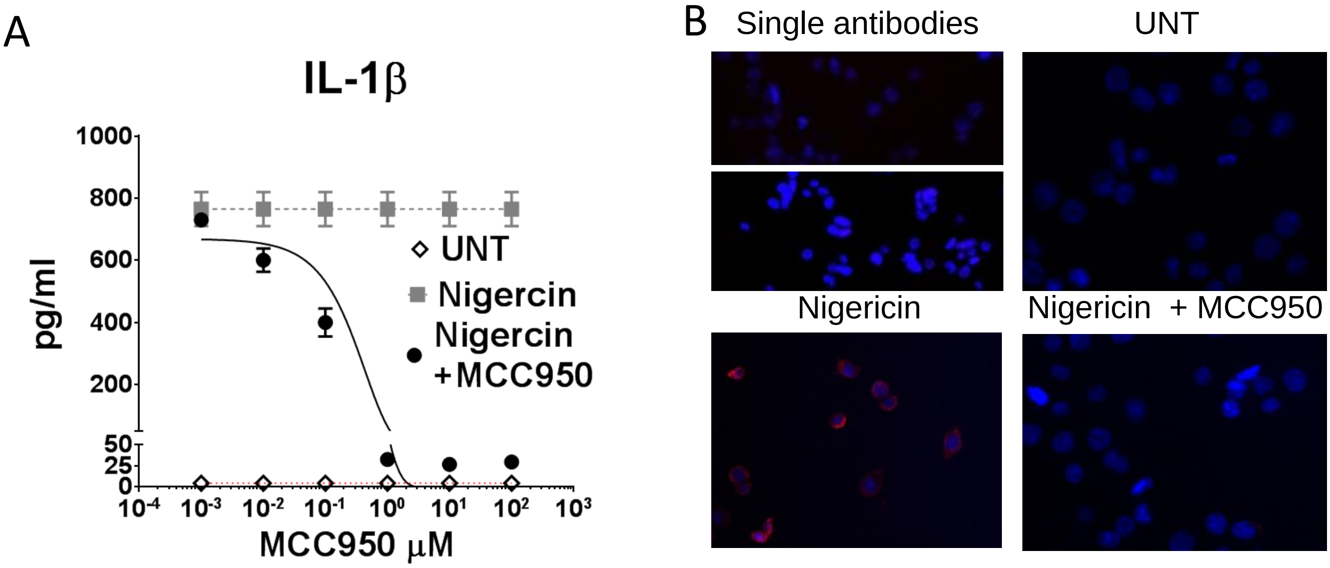

A. Release of IL-1β from PMA differentiated THP-1 cells after 24 h of treatment with nigercin and MCC950. **B.** Detection of NLRP3 inflammasome formation by Duolink proximity ligation assay, this assay stains ASC and NLRP3 in PMA differentiated THP1 cells. Blue is DAPI and red detects proximately of ASC and NLRP3 when in close proximity. Single Ab controls were treated with either anti-NLRP3 (top panel) or anti-ASC (bottom panel) primary Ab alone, and then using the same protocol of secondary Ab. THP-1 cells were treated with NLRP3 agonist nigercin at 5 µg/ml for 24 h with or without the presence of 1µM MCC950.

Public

Supplemental 3

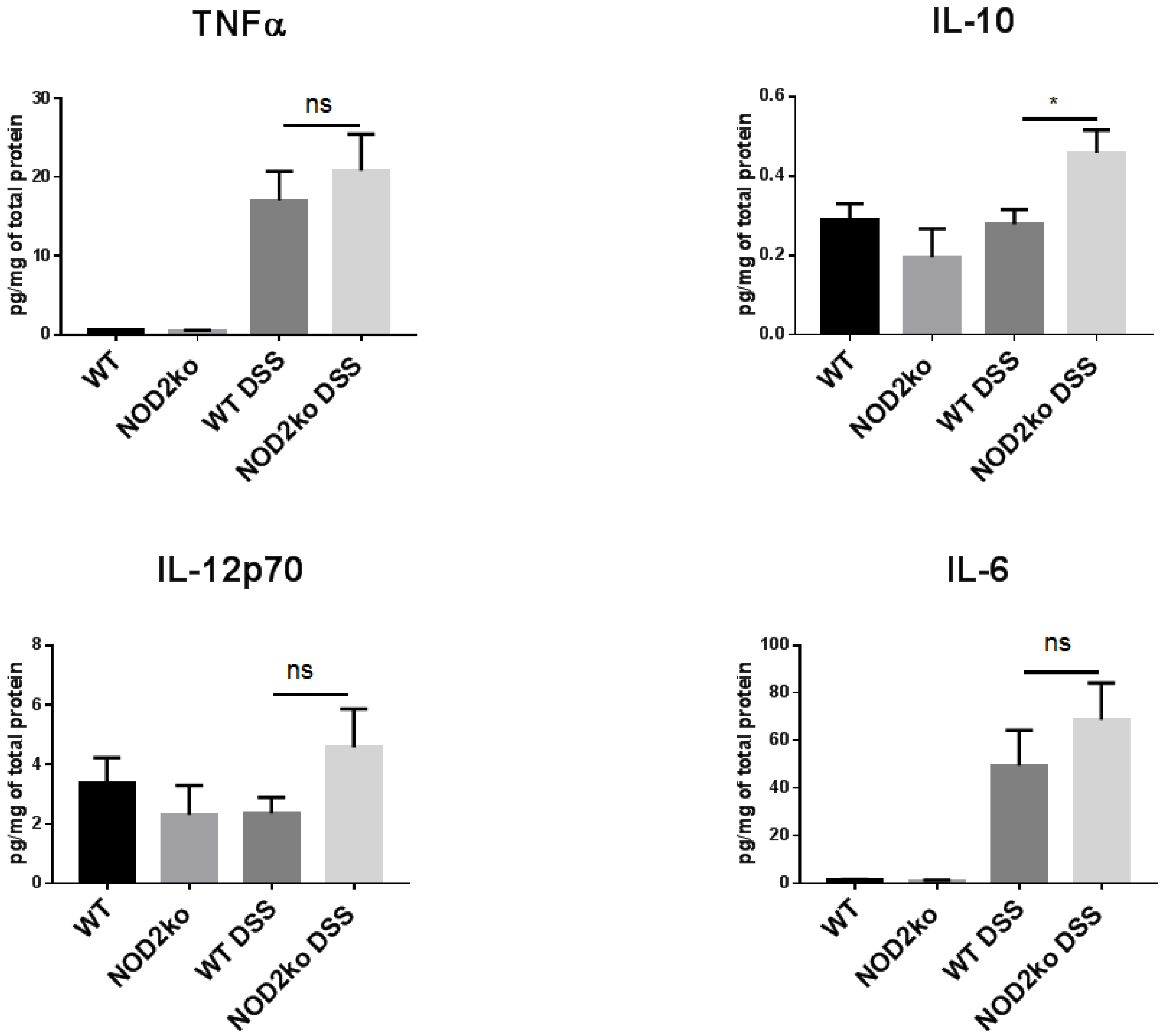

Concentrations of TNF-α, IL-10, IL-12p70 and IL-6 in colon homogenates divided by the total protein concentration of the colon homogenates from at least 8 mice per group.

Public

Supplemental 4

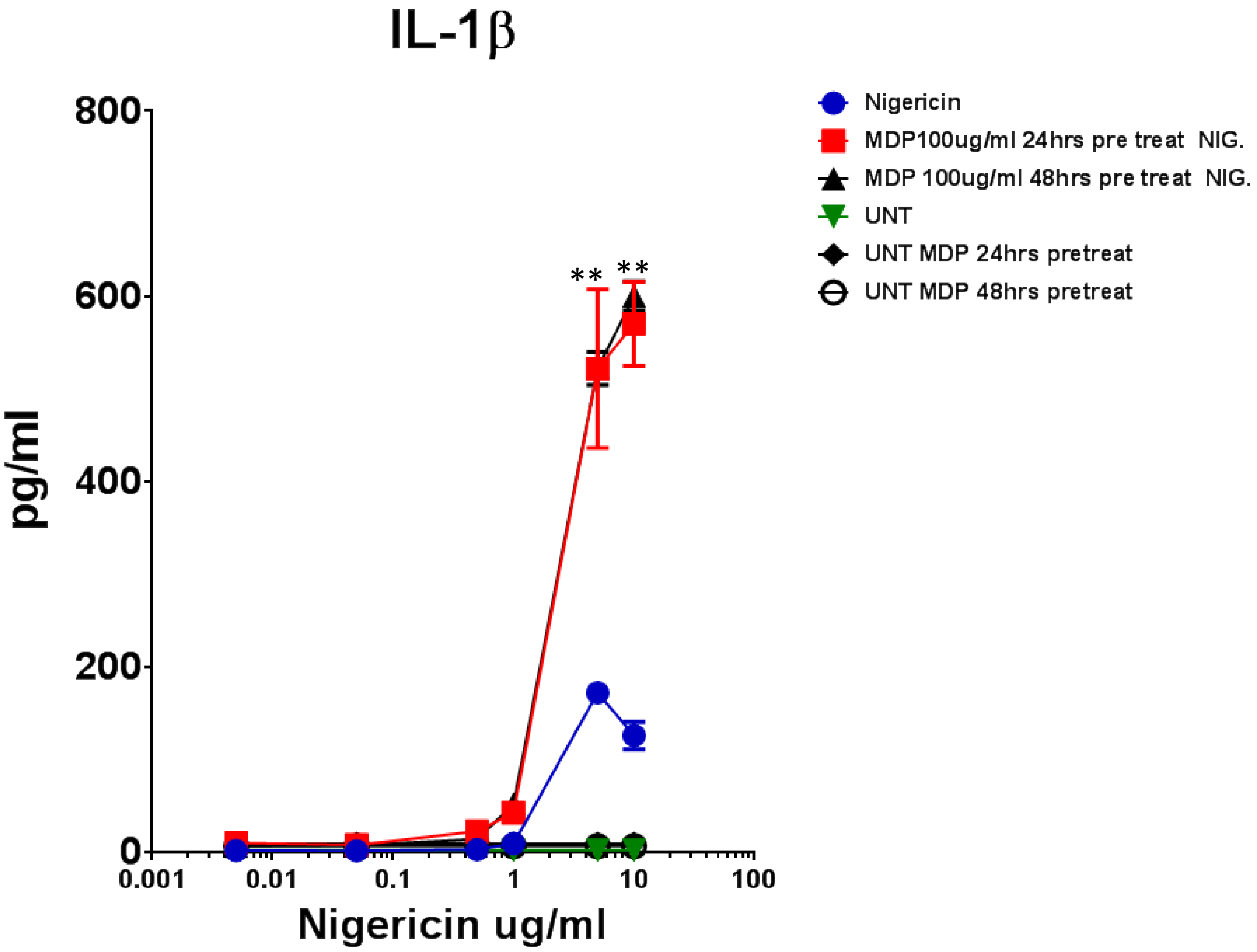

IL-1β concentrations from supernatants of PMA differentiated THP-1 cells. Cells were pretreated for 24 and 48 h with MDP at 100 µg/ml, followed by treatment with nigericin at the doses indicated.

Public

Supplemental Figure 5

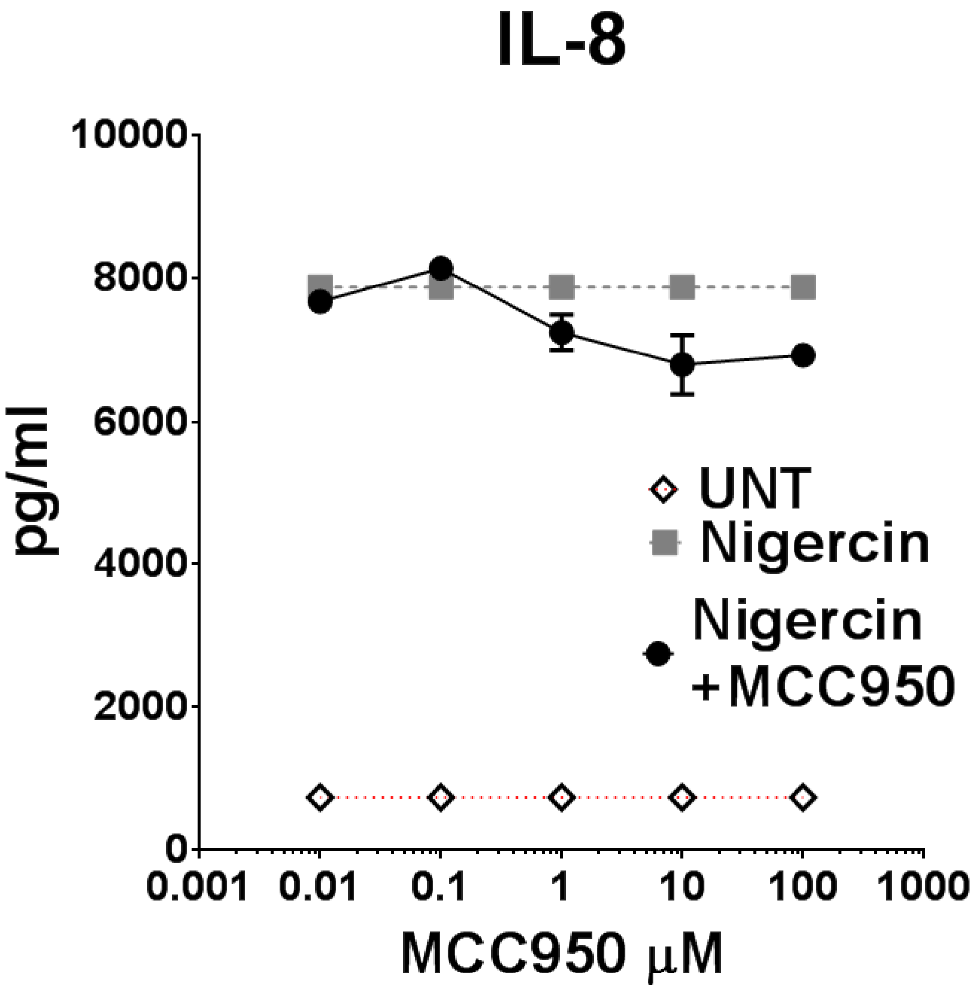

IL-8 from PMA differentiated THP-1 cells. Cells were treated with nigericin at 5 μg/ml and a concentration gradient of MCC950.

Public

Supplemental Figure 6

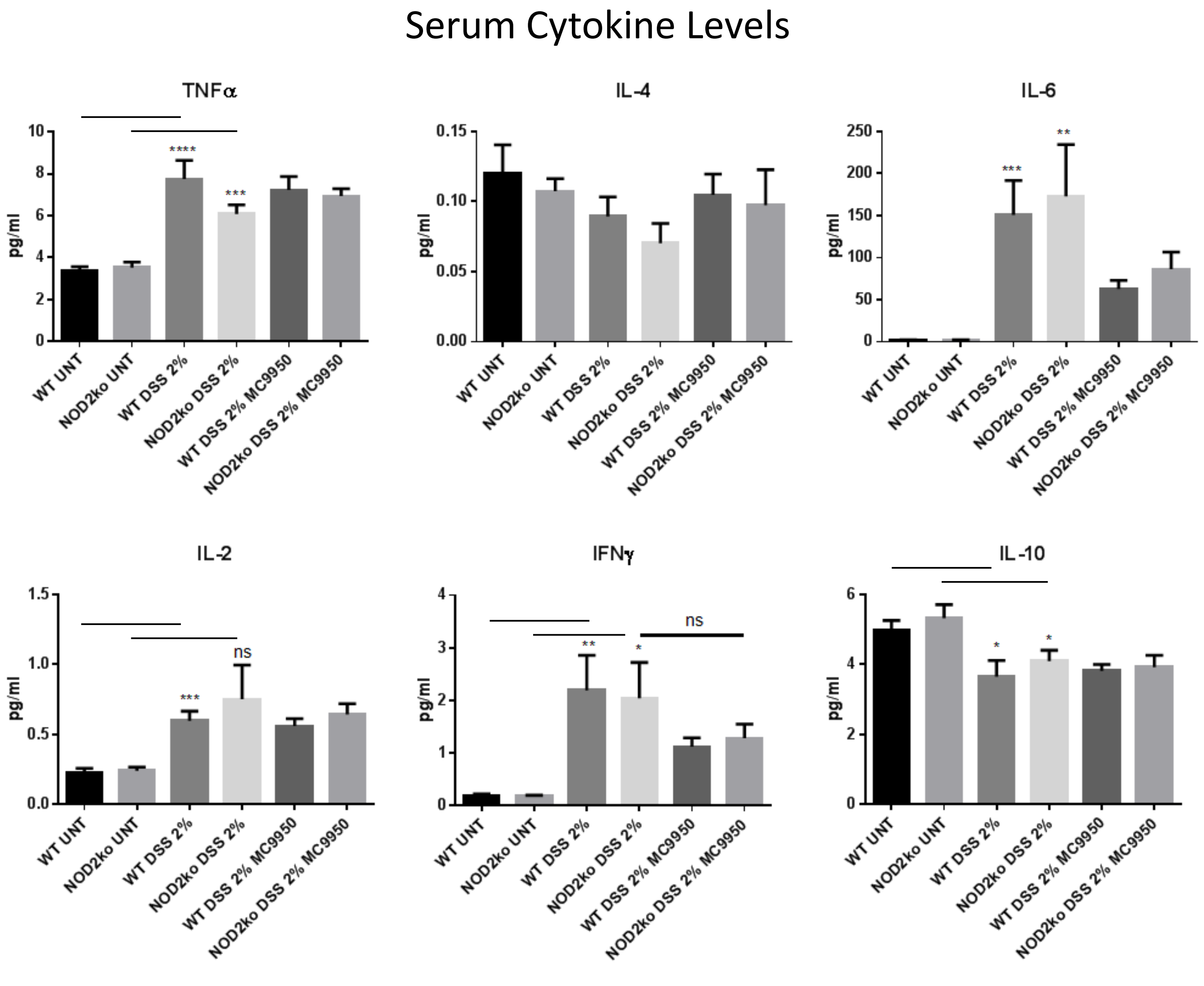

Concentrations of cytokines in the sera after 9 d of 2% DSS exposure.

Public

Supplemental Figure 7

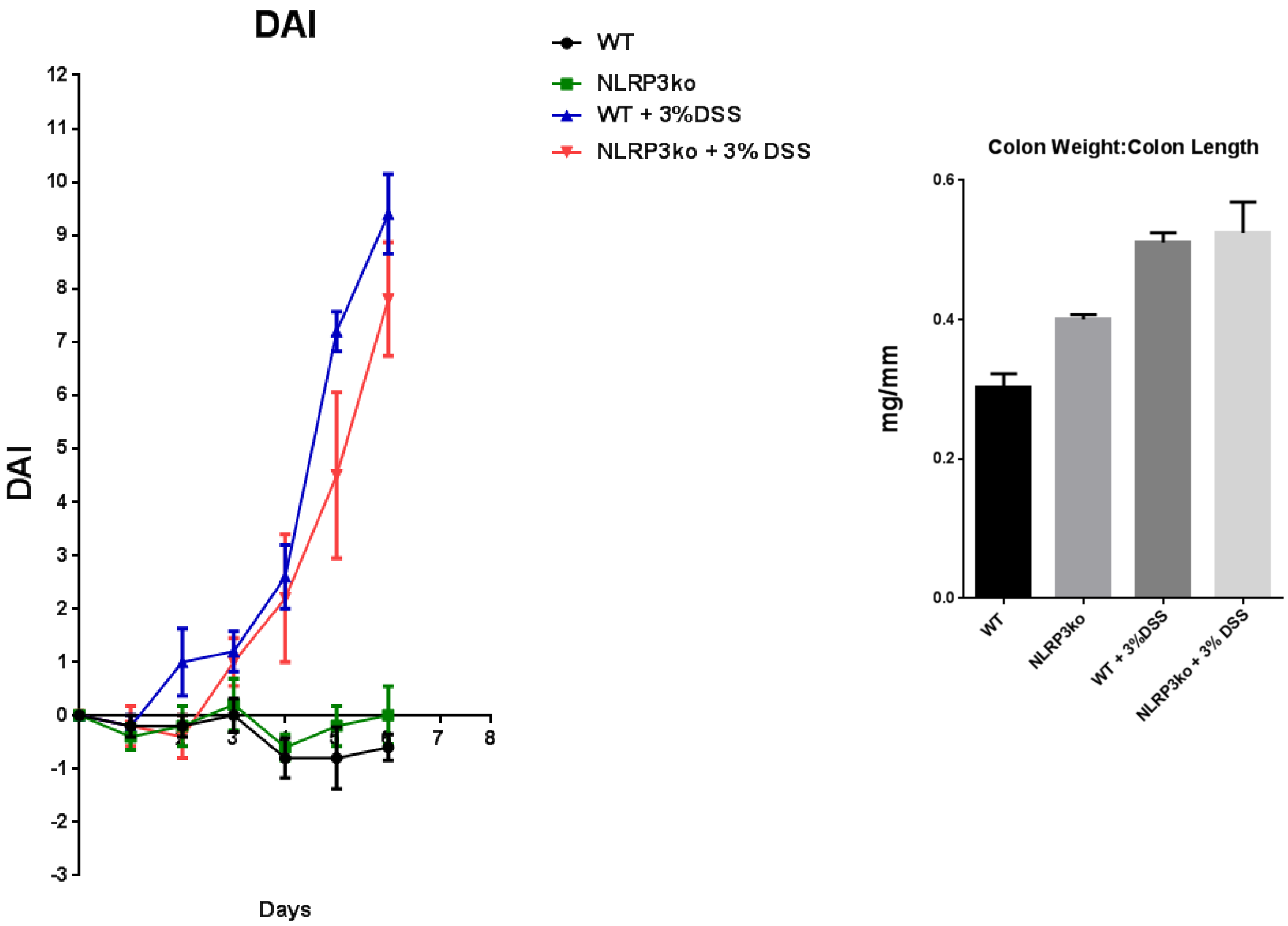

DSS at 3% in *Nlrp3* knockout mice. Disease Activity Index, scored by the addition of Stool consistency score, occult fecal blood score and body mass score. Colon masses (mg) divided by the length of colon (mm) on d 9 of DSS treatment.

Public

Supplemental 8

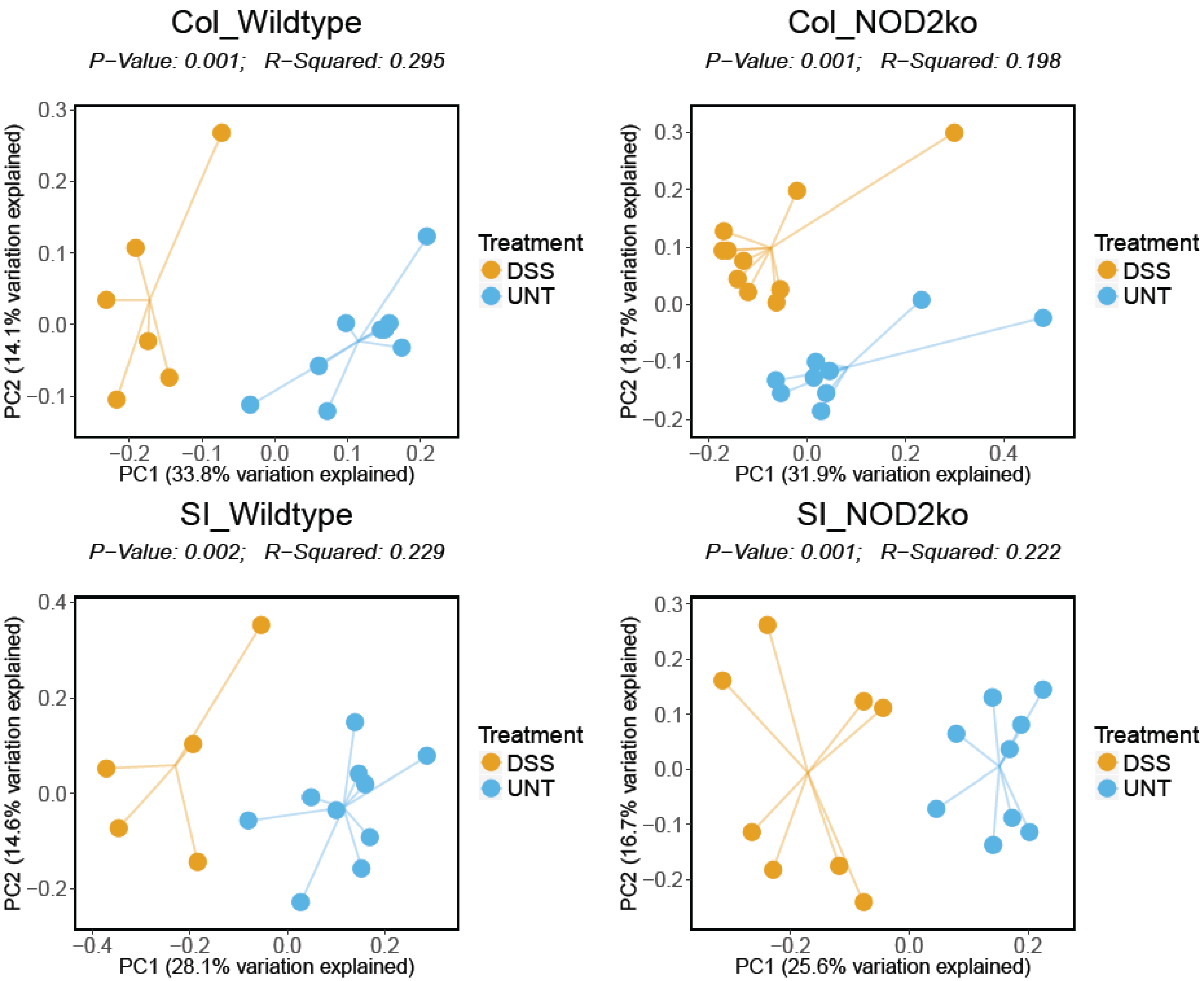

PcOA analysis using a un-weighted UniFrac of small intestinal and colonic bacteria in wild type and *Nod2-/-* mice comparing un-treated and 2% DSS treated mice.

Public

Supplemental Figure 9

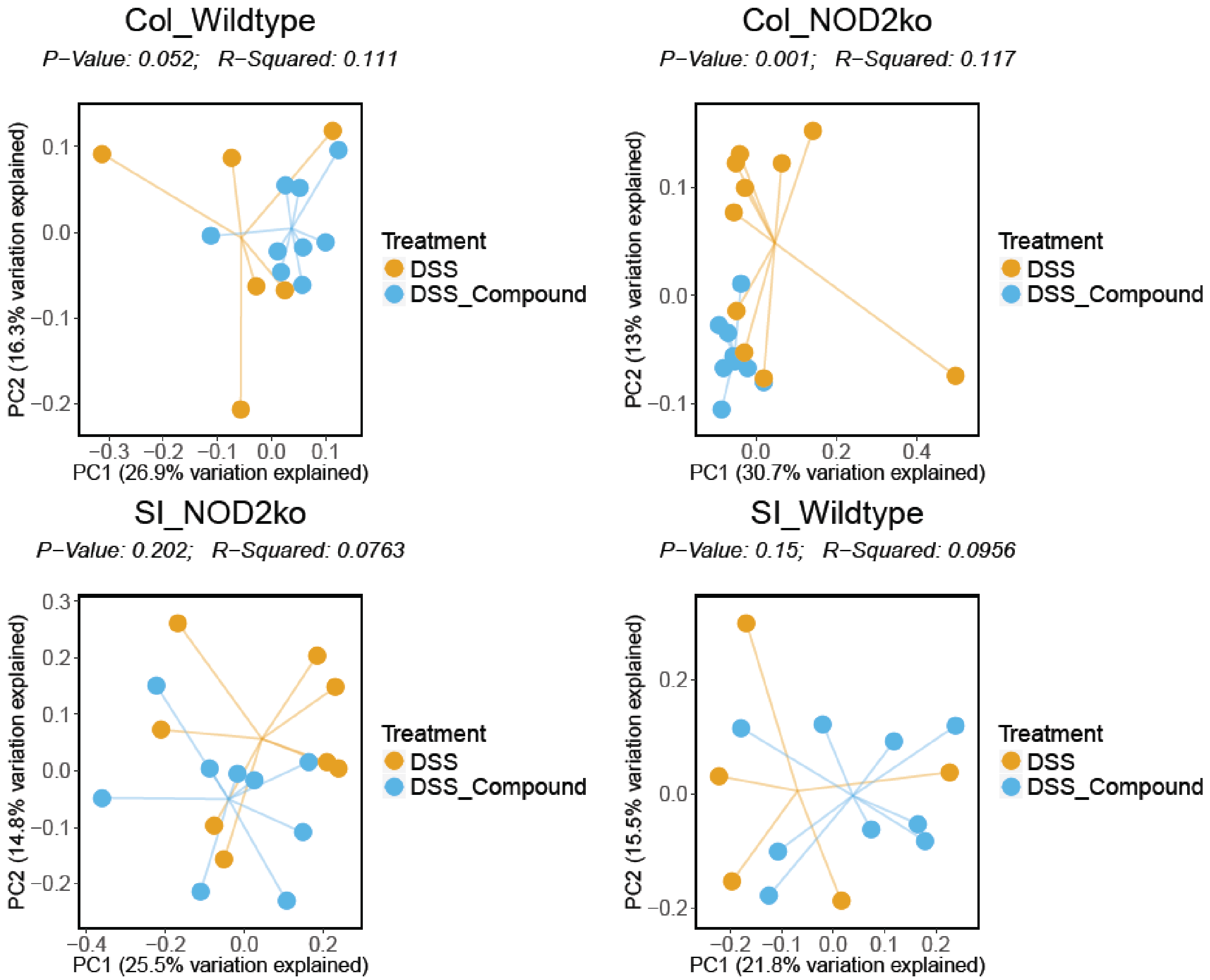

PcOA analysis using a un-weighted UniFrac of small intestinal and colonic bacteria in wild type and *Nod2-/-* mice comparing 2% DSS-treated mice and 2% DSS and MCC950-treated mice.

Public

Supplemental Figure 10

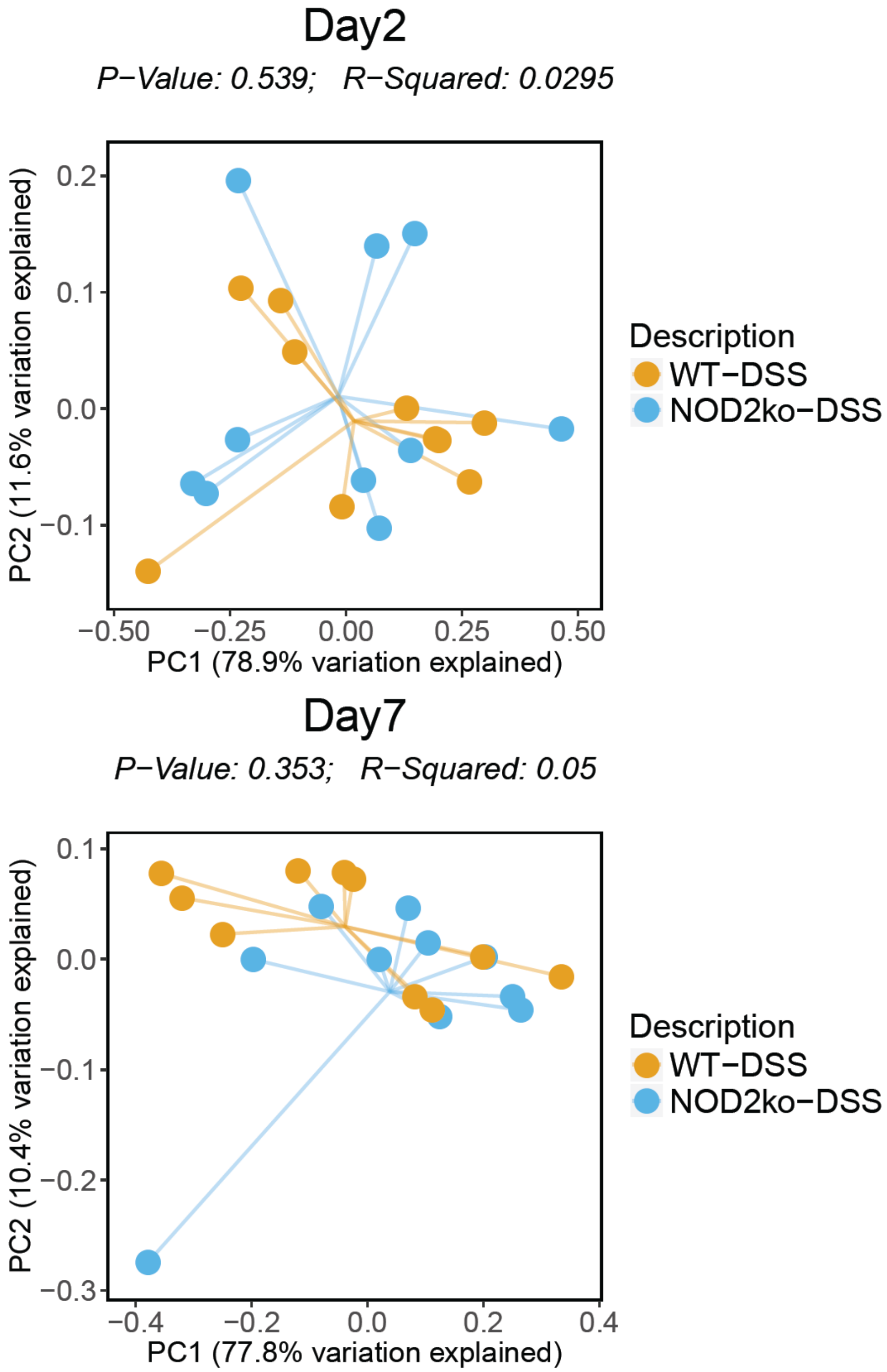

PcOA analysis using a un-weighted UniFrac in the feces in wild type and *Nod2-/-* mice treated with 2% DSS at d 2 and d 7 of DSS exposure.

Public

Supplemental Figure 11

Up-regulated

Tyk2
Tlr2
Ltb4r1
Ddx58
Tlr8
Il1rl2
Itgal
Mapk14
Irak3
Marco
Btk
Clec5a
Ppbp
Tnfsf10
Il12a
Ptpn6
Il13
Cd22
Tlr3
Fkbp5
Il18
Adal
Cd28
Il21r
Pdcd2
Ikzf1
Abcb10
Ifitm1
Itga2b
Il33
Cradd

Down-regulated

Fcgr4	Cd83	Il7r	C1qb
Cxcl1	Cxcl9	Tnfaip3	Cd14
Mx1	Fcgr1	Il6st	Ptafr
Mbp	Cfb	Ccl7	B2m
Cd82	H2-DMa	Stat5a	Pla2g2e
Tnfaip6	Hamp	Clu	Gpr183
Cd27	Cish	Il10	Mapk11
Vcam1	Cd80	Il15ra	Ccl22
Ccl2	Cxcr1	Mapkapk2	Ccl4
Slamf1	Ifi204	Nfkbiz	Bcl6
Ccl3	Csf1r	Pdgfb	Icosl
Il10ra	Ets1	Il15	Btla
Icam2	Stat4	Il1r1	Plau
Ccrl2	Tnfrsf8	Il1rap	Icam1
Ccl5	Nfkbia	H2-Aa	Tollip
Il1rn	Il27	Socs1	Csf2
Irf7	Il23a	Irf4	Notch1
Ifi35	Cd99	Cd74	Tnfrsf4
Ptgs2	Ccl12	H2-DMb2	
Tnfrsf9	Nfil3	Nfatc1	Ptger4
Tap1	Tnfrsf13b	Cd44	Fn1
Fcgr3	Entpd1	Cd109	
Il6	Cd86	Pou2f2	
Lta	H2-K1	Phlpp1	
Ctsc	Ifngr2	Nod2	
Ly86	Abcb1a	Pdcd1lg2	
Tnfrsf1b	Cd69	Cxcr4	
Socs3	Lif	Cxcl10	
Tgfbr1	Ptpn2	Ccl8	
Tlr9	Stat3	Smad3	
Plaur	H2-Eb1	Tnfsf15	
Cd274	Traf6	Fas	
Casp3	Litaf	Bcl3	
Bst2	Irak2	Cdh5	
Irf1	Cd40	Il2ra	
Traf1	Irf8	Ccr5	
Ctss	Il4ra	Map4k4	
Cdkn1a	Hif1a	H2-Q10	
Slamf7	Cebpb	Tagap	
Tnf	Ccr7	C1qa	

List of genes that are significantly higher and lower in *Nod2-/-* BMDM after treatment with LPS and nigericin compared with wild type BMDMs as found in a Nanostring mouse immunology panel.

Public
